# ChIPseek, a web-based analysis tool for ChIP data

**DOI:** 10.1186/1471-2164-15-539

**Published:** 2014-06-30

**Authors:** Ting-Wen Chen, Hsin-Pai Li, Chi-Ching Lee, Ruei-Chi Gan, Po-Jung Huang, Timothy H Wu, Cheng-Yang Lee, Yi-Feng Chang, Petrus Tang

**Affiliations:** Molecular Medicine Research Center, Chang Gung University, Taoyuan, Taiwan; Bioinformatics Center, Chang Gung University, Taoyuan, Taiwan; Graduate Institute of Biomedical Sciences, Chang Gung University, Taoyuan, Taiwan; Department of Microbiology and Immunology, Medical School of Chang Gung University, Taoyuan, Taiwan; Department of Biological Science and Technology, National Chiao Tung University, HsinChu, Taiwan; Institute of Biomedical Informatics, National Yang-Ming University, Taipei, Taiwan

**Keywords:** ChIP-seq, ChIP-chip, Analysis tool, Web-services, Peak annotation, Motif identification, Filter tools, Comparison

## Abstract

**Background:**

Chromatin is a dynamic but highly regulated structure. DNA-binding proteins such as transcription factors, epigenetic and chromatin modifiers are responsible for regulating specific gene expression pattern and may result in different phenotypes. To reveal the identity of the proteins associated with the specific region on DNA, chromatin immunoprecipitation (ChIP) is the most widely used technique. ChIP assay followed by next generation sequencing (ChIP-seq) or microarray (ChIP-chip) is often used to study patterns of protein-binding profiles in different cell types and in cancer samples on a genome-wide scale. However, only a limited number of bioinformatics tools are available for ChIP datasets analysis.

**Results:**

We present ChIPseek, a web-based tool for ChIP data analysis providing summary statistics in graphs and offering several commonly demanded analyses. ChIPseek can provide statistical summary of the dataset including histogram of peak length distribution, histogram of distances to the nearest transcription start site (TSS), and pie chart (or bar chart) of genomic locations for users to have a comprehensive view on the dataset for further analysis. For examining the potential functions of peaks, ChIPseek provides peak annotation, visualization of peak genomic location, motif identification, sequence extraction, and comparison between datasets. Beyond that, ChIPseek also offers users the flexibility to filter peaks and re-analyze the filtered subset of peaks. ChIPseek supports 20 different genome assemblies for 12 model organisms including human, mouse, rat, worm, fly, frog, zebrafish, chicken, yeast, fission yeast, *Arabidopsis*, and rice. We use demo datasets to demonstrate the usage and intuitive user interface of ChIPseek.

**Conclusions:**

ChIPseek provides a user-friendly interface for biologists to analyze large-scale ChIP data without requiring any programing skills. All the results and figures produced by ChIPseek can be downloaded for further analysis. The analysis tools built into ChIPseek, especially the ones for selecting and examine a subset of peaks from ChIP data, provides invaluable helps for exploring the high through-put data from either ChIP-seq or ChIP-chip. ChIPseek is freely available at http://chipseek.cgu.edu.tw.

## Background

The chromatin immunoprecipitation (ChIP) assay is a powerful technique to examine the specific interaction between proteins and DNA within living cells [1–3]. The DNA-binding proteins play important roles in regulating many cellular processes including gene expression, replication, recombination, repair, methylation and chromatin remodeling. The ChIP assay provides comprehensive analysis to reveal specific DNA and protein interaction according to: modifications on DNA-binding proteins (phosphorylation, acetylation, methylation, ribosylation and ubiquitination); DNA methylation; spatial and temporal regulation during development; different cell types and physiological conditions. Application of ChIP assay is therefore versatile, specific applications are listed as follows: identifying the transcription factor binding sites [4], locating the sites of histone modifications [5], mapping the hypermethylated DNA region in stem cells [6], identifying the changes in epigenetic modifications according to different developmental stages [7], and identifying the epigenetic changes in different cell types [8] and in cancers [5, 9].

To perform ChIP assay, proteins and DNA are in vivo-crosslinked by formaldehyde, chromatin is sonicated into small fragments, and an antibody is used to immunoprecipitate the protein-DNA complexes [1]. The DNA fragments are further purified and subjected to various analyses, for instance, PCR, cloning, hybridization and sequencing. The ChIP assay not only allows quantitative detection of a given protein on a particular DNA site but also permits the mapping of genome-wide DNA-protein interaction. With the advancement of sequencing technology, ChIP assay has been adapted to use high throughput sequencing (ChIP-seq) for mapping the DNA region or even defining the consensus sequences that are involved in DNA-protein interaction [10–13]. Several peak calling tools have been proposed for ChIP-seq data analysis, such as MACS, PeakSeq and HPeak [14–16]. However, a major challenge comes after the ChIP-seq—processing, analyzing and presenting the ChIP-seq data. Several tools have been developed for ChIP-data analysis. For instance, CisGenome provides analysis and visualization of ChIP-data [17]; Hypergeometric Optimization of Motif EnRichment (HOMER) can find motifs from peak files and annotated peaks [18]; *Po*sitioning *d*ata*b*ase and *a*nalysis *t*ool (Podbat) provides analysis and visualization of peaks [19]; Cistrome contains many ChIP data analysis packages [20]; EpiExplorer offers an interactive web interface for visualization of peak distribution [21]; Genomic Association Test (GAT) can test whether multiple sets of peaks significantly overlap with each other [22]; PscanChIP can identify the enriched transcription factor binding sites [23]; and PAVIS focuses on annotation and visualization of peaks [24]. A summary of these tools is shown in Table 1.

**Table 1:** **Summary of available tools**

	CisGenome [17]	BEDTool [25]	HOMER [18]	Podbat [19]	Cistrome [20]	EpiExplorer [21]	GAT [22]	PscanChIP [23]	PAVIS [24]	ChIPseek
Web-based analysis tool				✓	✓	✓		✓	✓	✓
Graphic user interface	✓			✓	✓	✓		✓	✓	✓
Annotation	✓		✓	✓	✓	✓		✓	✓	✓
Plot of position relative to TSS	✓			✓	✓	✓		✓		✓
Plot of peak length distribution						✓				✓
Filter (by peak length)						✓				✓
Filter (by position relative to TSS)										✓
Post-filter analysis										✓
Motif identification	✓		✓		✓			✓		✓
Comparison	✓	✓		✓	✓	✓^+^	✓			✓
Link to UCSC genome browser	✓	✓			✓	✓		✓		✓
Plot of genomic location	✓			✓	✓				✓	✓
Sequence extraction		✓								✓
Built-in peak databases	✓					✓				✓
Enrichment analysis				✓	✓		✓	✓	✓	

^+^EpiExplorer provides comparison between an uploaded file and a built-in database.

As shown in Table 1, most of the existing tools do not have built-in genomic location features for comparison. There are already many large-scale projects that focus on ChIP-seq of transcription factors and histone modifications, such as the ENCODE and modENCODE projects [26, 27]. The ENCODE consortia have already completed ChIP-seq for 161 transcription factors in more than 100 cell types using the same guidelines and quality-controls [27]. These databases are highly valuable and very informative, especially in terms of their breadth and quality. Yet most of these tools do not provide the functionality to filter based on peak length or on position relative to a transcription start site (TSS). However, after obtaining a list of peaks from either ChIP-seq or ChIP-chip, researchers want to obtain an overview of all peaks and then pick out the probable or significant peaks for further validation and investigation. The common procedure to obtain the candidate peaks is to filter and then re-analyze the subset of data. To exploit the well-established databases and provide the functionality to filter peaks, we present a web-based analysis tool, ChIPseek, which can (1) annotate peak files; (2) filter datasets based on the size of peaks; (3) filter datasets based on the distance to the nearest TSS; (4) identify enriched motifs based on uploaded datasets or filtered datasets; (5) compare two sets of peaks; (6) extract peak sequences and motif identification; (7) compare an uploaded dataset or filtered dataset with built-in datasets; (8) plot peaks on chromosome ideograms, and (9) select peaks based on their closest genes corresponding to the gene ontology (GO) or signaling pathways (KEGG database) [28, 29].

ChIPseek takes BED, GFF and text files as input and provides annotation for the peaks. In addition to peak annotation, ChIPseek provides pie charts, bar charts and histograms for peak location distribution, which offer an intuitive way to understand the distribution of all peaks. ChIPseek also generates histograms indicating the frequency distribution of the peak length and the distance to the nearest TSS. All these figures or tables generated by ChIPseek can be downloaded for further analysis. Currently, ChIPseek supports data from *Homo sapiens*, *Mus musculus*, *Rattus norvegicus*, *Caenorhabditis elegans*, *Drosophila melanogaster*, *Xenopus tropicalis*, *Danio rerio*, *Gallus gallus*, *Saccharomyces cerevisiae*, *Schizosaccharomyces pombe*, *Arabidopsis thaliana* and *Oryza sativa*. Overall, ChIPseek provides both systematic visualization and filter tools for ChIP data analysis through a user-friendly interface. We believe that ChIPseek is a useful and valuable tool for further ChIP and epigenetic studies.

## Implementation

ChIPseek is mainly constructed using python and the website is written in mod-python, Google Charts, jQuery, JavaScript and PHP [30–32]. ChIPseek also integrates several well-developed and widely used tools, such as HOMER and BEDTools [18, 25], to provide better peak annotation and analysis.

### Annotation table

The first result generated by ChIPseek is an annotation table. After uploading a file(s), ChIPseek first checks for file format. If the uploaded file(s) contains the proper location information, then ChIPseek can further utilize annotatePeaks.pl from HOMER [18] to annotate the peaks according to their genomic locations.

### Links to the UCSC Genome browser

In the annotation table, ChIPseek provides a link to the UCSC genome browser to allow interactive access to the genomic sequence for each peak. ChIPseek also adds custom tracks of uploaded files on the UCSC website. To add custom tracks, ChIPseek incorporates BEDTools [25] and two tools downloaded from UCSC ftp [33, 34]. ChIPseek first uses fetchChromSizes to produce a file of recorded sizes of chromosomes belonging to the organism of interest. ChIPseek then uses mergeBed and sortBed to ensure that the input peak file is compatible with further conversion. Finally, ChIPseek employs bedGraphToBigWig to convert the peak file, together with chromosome size information, into bigWig file. The input for bedGraphToBigWig must be non-redundant and sorted by position. ChIPseek utilizes shell scripts to prepare a proper input BED file for bedGraphToBigWig. The bigWig file can then be uploaded to UCSC to visualize the peak information. If users upload multiple peak files, then ChIPseek can generate multiple tracks and display them simultaneously in the UCSC genome browser.

### Statistics of peaks

ChIPseek generates several distribution figures based on the annotation results. These plots are generated in real time by python scripts, mod_python and JavaScript libraries from Google charts [30].

### Filter criteria

In order to provide a comprehensive analysis of the distribution and location of the protein binding sites/peaks relative to TSS, and to classify the biological impact of the targets to a specific group of gene in the same metabolic or signal pathway, users can further subdivide the peaks by using six filter criteria based on (a) Distance to nearest TSS, (b) Peak length, (c) Peak location, (d) Gene list, (e) Gene Ontology (GO), and (f) Kyoto Encyclopedia of Genes and Genomes (KEGG) pathway. ChIPseek uses JavaScript in filter criteria (a) Distance to TSS and (b) Peak length to provide slider bars and text boxes for users to specify a range (bp) to filter the peaks according to distance to TSS or peak length. ChIPseek then generates histogram with mod_python and Google chart libraries for the filtered subset of peaks. For filter criteria (e) GO and (f) KEGG, the relationships between GO terms are downloaded from the GO website (http://www.geneontology.org). The functional annotations of KEGG are downloaded from KEGG BRITE database (http://www.genome.jp/kegg/brite.html). KEGG ID conversion API is used to convert the NCBI gene ID and KEGG Orthology (KO) ID. Several in-house scripts are used to integrate all functional hierarchies into tree structures. Fancytree from jQuery Plugin Registry is used to show the relationship between those functional annotation terms [35].

### Get genomic sequences

ChIPseek employs fastaFromBed to extract corresponding genomic sequences from the corresponding genome. The output FASTA files are converted into tab-delimited text files with in-house python scripts.

### Motif identification

For enriched motif identification, ChIPseek integrates findMotifsGenome.pl, a tool from HOMER [18] which can identify enriched motifs in genomic regions, and use Weblogo and Ghostscript for sequence logo generation [35, 36]. The motif identification includes the following steps: extraction of sequences, background selection, GC-content normalization (for background sequences), and finally motif discovery (including novel motifs and known motifs). ChIPseek provides four options (±25 bp, ±100 bp, ±250 bp and ±500 bp) for users to specify the size of the region to search for motifs.

### Chromosome ideograms

To achieve a better understanding of overall peak distribution in the genome, ChIPseek provides ideograms of chromosomes indicating the relative positions of the peaks associated with them. The ideograms are implemented in JavaScript, and we adapted the ideogram plotting code Ideogram Viewer from MD Anderson Center to ChIPseek [37]. We also made several modifications: (1) we added options for different colors to mark the peaks on a chromosome; (2) we added a hyperlink to each mark and provide a link to the UCSC genome browser; (3) we changed the content that is displayed when the mouse pointer hovers over an item; and (4) we changed the ends of the chromosome into round ends. The cytoband information used in ideograms is downloaded from UCSC [38].

### Intersection of peaks

ChIPseek provides the option to compare two set of peaks. The comparison generates the relationship between peaks from the two sets. To obtain these peak relationships, ChIPseek employs several in-house scripts, as well as intersectBed and coverageBed from BEDdtools [25].

### Database of transcription factor binding sites

ChIPseek also provides a binding sites database of transcription factors for human genome assemblies (hg19, hg18 and hg17). This database is generated from the Encyclopedia of DNA Elements (ENCODE) project [26], which contains 161 transcription factors in its database. The latest ENCODE dataset (hg19) is obtained from the UCSC Genome Browser download server. We further applied liftOver [39] to cover the binding site to obtain corresponding coordinates for hg18 and hg17. All of these databases are available in the comparison section.

## Results and discussion

ChIPseek is a web-based tool designed for ChIP data analysis. This software includes the following features: annotation of peaks; motif identification; peak sequence extraction; peak plotting on chromosome ideograms; generation of histograms of the peak length distribution; generation of histograms of the distance to the nearest TSS; generation of pie charts of the genomic location, and peak filtering by peak length, distance to the nearest TSS, peak location, gene list, GO categories, and KEGG pathways, etc. Currently, ChIPseek mainly focuses on the human genome (hg17, hg18 and hg19) but also provides most of the analytic functions for other model organisms, such as mice (mm8, mm9 and mm10), rats (rn4 and rn5), worms (ce6 and ce10), flies (dm3), frogs (xenTro3, xenTro2), zebrafish (danRer7), chickens (galGal4), yeast (sacCer2 and sacCer3), *Schizosaccharomyces pombe* (ASM294v1), Arabidopsis (tair10) and rice (*Oryza sativa*, msu6). ChIPseek supports three types of input file formats: text file, BED format and GFF format. Users can upload their own data to perform all supported analyses through the web interface. Here, we demonstrate the usage and analysis functions of ChIPseek with two datasets: (1) the DNA binding sites of four transcriptional activators, ATF2, ATF3, ETS1 and GATA1, and (2) binding sites of the transcriptional repressor CTCF.

### Demo dataset 1: Transcription factor binding sites of ATF2, ATF3, ETS1 and GATA1

**Figure 1:**
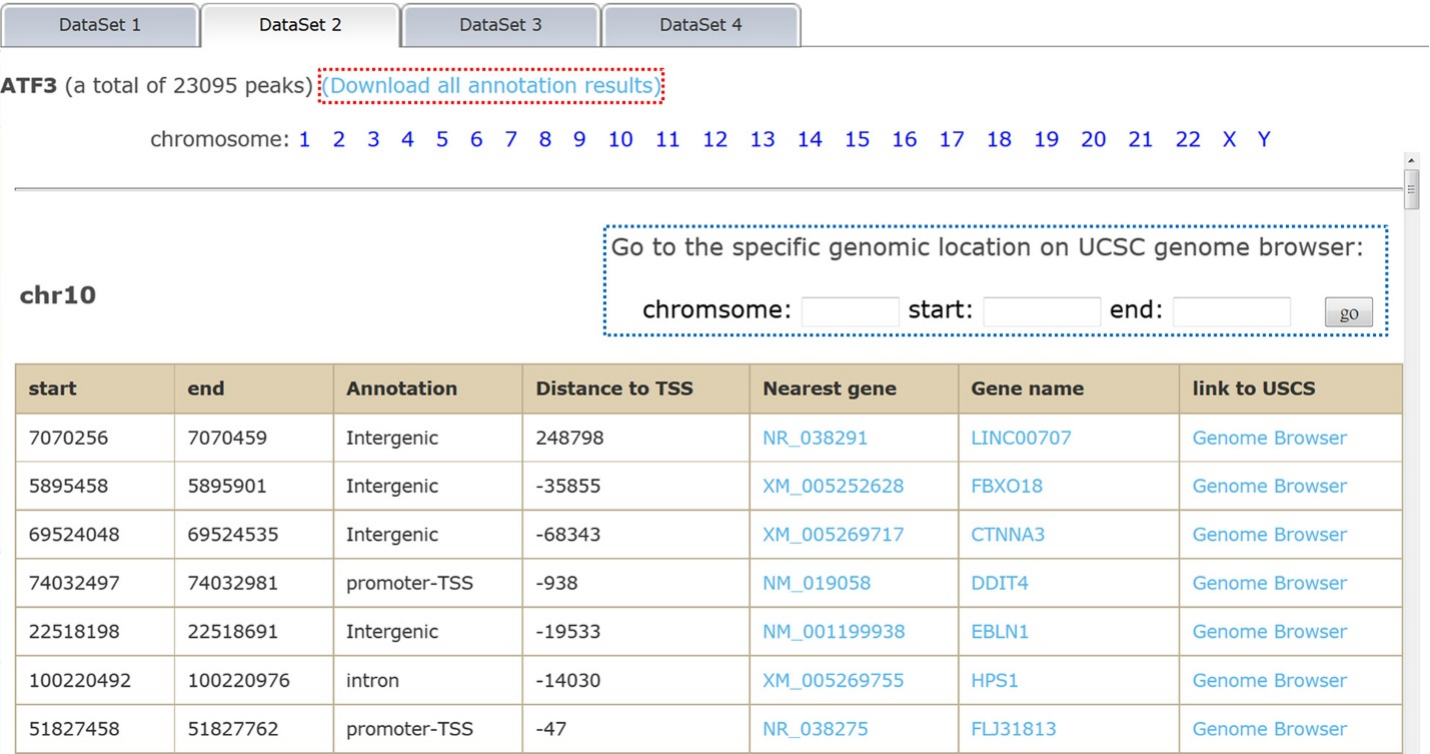
**Annotation results for binding sites of ATF2, ATF3, ETS1 and GATA1.** ChIPseek separates annotation tables into separate tabs for each upload file. In each tab, ChIPseek shows annotation results for each chromosome in an annotation table. Here, a partial annotation result for binding sites of ATF3 in chromosome 13 is shown. For this TF, the total number of peaks (23,095 peaks) is shown above the annotation table followed by a link for downloading the full annotation table (highlighted by the red box). Within the table, each column shows the location of peaks, genomics location annotation, distance to the nearest TSS, nearest RefSeq, gene name etc. The user can click on the title of each column to sort that column. In the table, words in light blue are hyperlinks leading to external databases or a genome browser, i.e., NCBI RefSeq database, UniProt database and the UCSC genome browser, for each peak. User may also specify the regions of interest and visit those particular regions using the text boxes above the annotation table (highlighted by the blue box).

The binding sites of transcriptional factors ATF2, ATF3, ETS1 and GATA1 are generated by the ENCODE project. We downloaded the BED files from the UCSC download server and used them as a demo dataset for ChIPseek. We uploaded four BED files and selected Human/hg19 as our genome assembly version from the multiple file upload page. The input files are checked and then annotated by ChIPseek. The results are shown in the annotation tables (as shown in Figure 1). If users upload multiple files, then the annotation tables for each file are listed in separate tabs. The annotation results are separated according to different chromosomes. The annotation table for each chromosome indicates start, end, location annotation, distance to the nearest TSS, nearest RefSeq, nearest gene name and link to the UCSC genome browser. Users can also link to external databases, NCBI and UniProt [40, 41], by clicking on the RefSeq or gene symbol. The total number of peaks is shown on top of each tab. In our demo samples, there are a total of 23095, 26026, 13737 and 20310 peaks for ATF2, ATF3, ETS1 and GATA1, respectively. Due to limited space on the browser, seven columns of peak information are displayed. The detailed information of the peaks can be downloaded by clicking the hyperlink “Download all annotation results” on the top-left corner within each separate tab. The full annotation results include the distance to the nearest TSS, nearest promoter ID, Entrez Gene ID, nearest Uniqene ID, nearest Refseq, nearest Ensembl gene name, gene symbol, gene aliases, gene description, genomic location annotation, and hyperlinks to external databases in text format.

ChIPseek provides links to the UCSC genome browser and displays uploaded peak data on the genome browser. UCSC integrates many biological information sources such as SNP, expression profile and regulation elements [34, 38], allowing users to access and explore peaks of interest at many levels. In the last column of the annotation table, ChIPseek provides a hyperlink for each peak, which can lead users to the corresponding region for that particular peak. By default, the region extends 1,000 bp upstream and downstream. Users can directly select a peak and explore other biological feature tracks in adjacent regions on the UCSC genome browser. ChIPseek also provides the flexibility for users to explore a specific region of interest with the genome browser. As shown in Figure 1, users can enter a specified region in the text boxes above the annotation table, click on the ‘go’ button, and be linked to the UCSC genome browser, focusing on that particular region in a new window.

**Figure 2:**
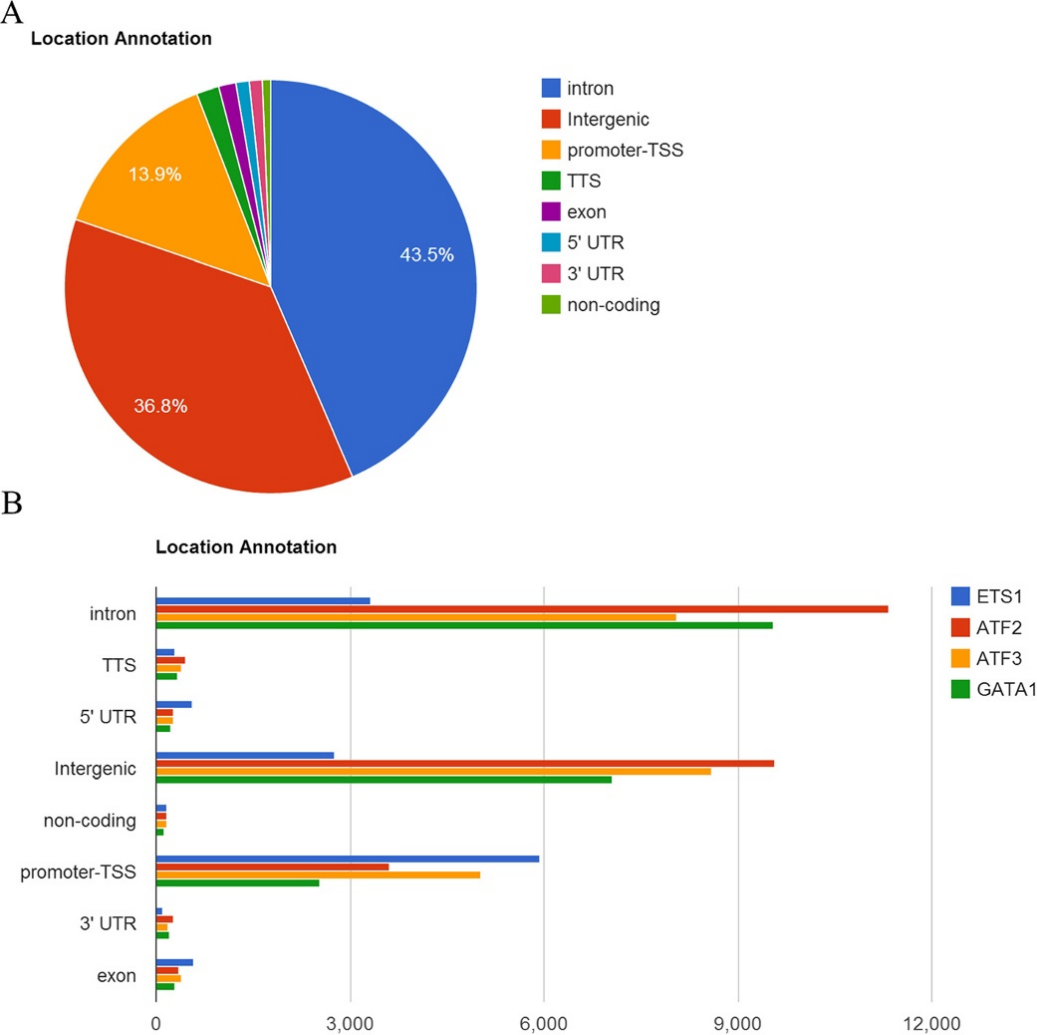
**Pie chart and bar chart of genomic location distribution. (A)** Pie chart of the genomic location distribution of transcription factor ATF2. This plot shows the percentage for each genomic location category. The categories are sorted by descending percentage. The exact percentage for each category appears if the mouse pointer hovers over a pie slice. **(B)** Bar chart of the genomic location distribution of four transcription factors, ETS1, ATF2, ATF3 and GATA1. All uploaded files are combined into the same bar chart. This bar chart reveals the actual number of peaks for each category when the mouse pointer hovers over each bar.

**Figure 3:**
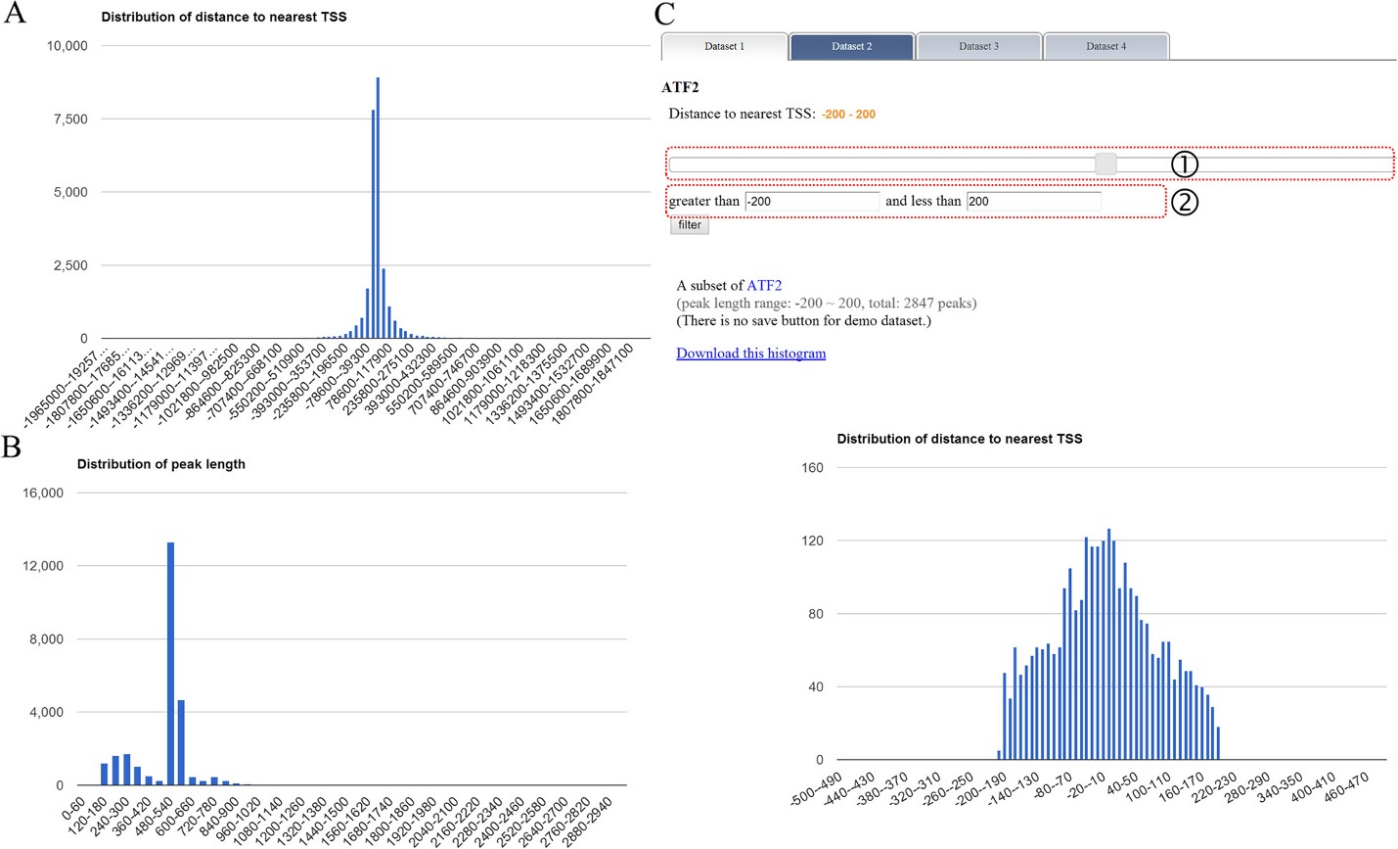
**Histogram of distance to the nearest TSS and of peak lengths. (A)** The distribution of distance to the nearest TSS. This example is of the transcription factor binding sites for ATF2. The x-axis of the histogram is centered at 0 and divided into 100 bins that cover the largest and smallest values of distance. As shown in this histogram, most of the binding sites are located near the TSS. The exact number of peaks for each range of distance appears when the mouse pointer hovers over that bar. **(B)** The distribution of the peak lengths of transcription factor binding sites of ATF2. Most of the peaks have a length smaller than 600 bp. Again, if users are interested in the exact number of peaks within each range, hovering over that range will reveal the value. **(C)** The user may use filter criteria to select a subset of peaks. There are two ways to filter the peaks (highlighted by the red boxes). The first is the slider bar and the second is the text box. In this example, we use text boxes to filter out peaks with distance to the nearest TSS larger than +200 or smaller than -200. After this operation, 2,847 peaks are left. After the selection step, the histogram is refreshed with this subset of peaks in real time. After this filter step, we save the filtered subset with the “save” button above the histogram.

The scores (height) of custom tracks indicated in the UCSC genome browser are either from users’ uploaded files or given by ChIPseek. If users uploaded BED files, GFF files or text files with score information, then the score will be shown on the UCSC genome browser. If there is no score information in the uploaded text file, ChIPseek will assign all peaks a score of 1. In that case, all peaks will have the same height on the UCSC genome browser. Moreover, the peaks in a single track on the UCSC genome browser cannot overlap. Therefore, if there are overlapping peaks in a single uploaded file, ChIPseek will merge those overlapping peaks and use the average score as a representation for the final merged peak.To visualize the peak distribution within the genome, the examination of location distribution is usually the first step in analyzing ChIP data. The location represents where each peak located and the value includes the 3′UTR (untranslated region), 5′UTR, exon, intergenic region, intron, non-coding, promoter-TSS and transcription termination site (TTS). Both pie charts and bar charts for visualizing the overall peak distribution are available in ChIPseek. If users upload multiple files, separate pie charts for each dataset and a bar chart containing all datasets for comparison are presented as shown in Figure 2. Users may choose a suitable chart to present their data. If users are interested in the detailed composition of each category, then the raw data of genomic location is also available in the full annotation table as mentioned previously.To display the frequency and distribution of the distance to the nearest TSS or distribution of peak lengths, histograms are used. As shown in Figure 3, the x-axis for the histogram of distance to the nearest TSS is centered at 0 and divided into 100 bins. The x-axis for the histogram of the length distribution starts at 0 and is divided into 50 bins. One of the unique features of ChIPseek is that it offers six filters allowing users to obtain a subset of peaks for further analysis. These six filters are established based on the following criteria: distance to the nearest TSS, lengths of peaks, genomic location, uploaded gene list, selected GO terms, and KEGG groups. Because the transcription factors are likely to be located near the TSS of regulated genes, we can filter the binding sites of ATF2 and ATF3 based on the “Distance to the nearest TSS”. Using this filter criterion, only the peaks located between -200 and +200 bp of the nearest TSS are selected. After this step, a total of 2,847 out of 26,026 ATF2 peaks and 3,999 out of 23,095 ATF3 peaks fit this criterion. A new histogram of the subset peaks in real time as shown in Figure 3. Two new files containing the two subsets of peaks will be generated by and saved in ChIPseek, and will be automatically named ATF2_-200-200_TSS and ATF3_-200-200_TSS, respectively. The naming system starts with the original uploaded file name, followed by the range and then “TSS”, which shows the filter criteria based on the distance to TSS. These two saved files can be used in the subsequent analysis.

In addition t.o generating plots for exploring the properties of peaks, ChIPseek also extracts the sequences of the peaks. These sequences can be used for further analysis such as TFsearch, DMEME, CentriMo or other ChIP data analysis tools etc. [42–45]. After clicking the “Peak sequences” on the menu, users can obtain a table of partial extracted sequences. The extracted sequences are presented in two forms: FASTA format and tab-delimited text format. The tab-delimited format contains 5 columns: chromosome, start position, end position, length of sequences and the DNA sequences. Our tools can also extract sequences for the subsets of uploaded files. As we already saved a subset of peaks (ATF2_-200-200_TSS and ATF3_-200-200_TSS) in the previous step, we can retrieve the sequences for these two saved subsets by clicking on “Get peak sequences” on the menu.

**Figure 4:**
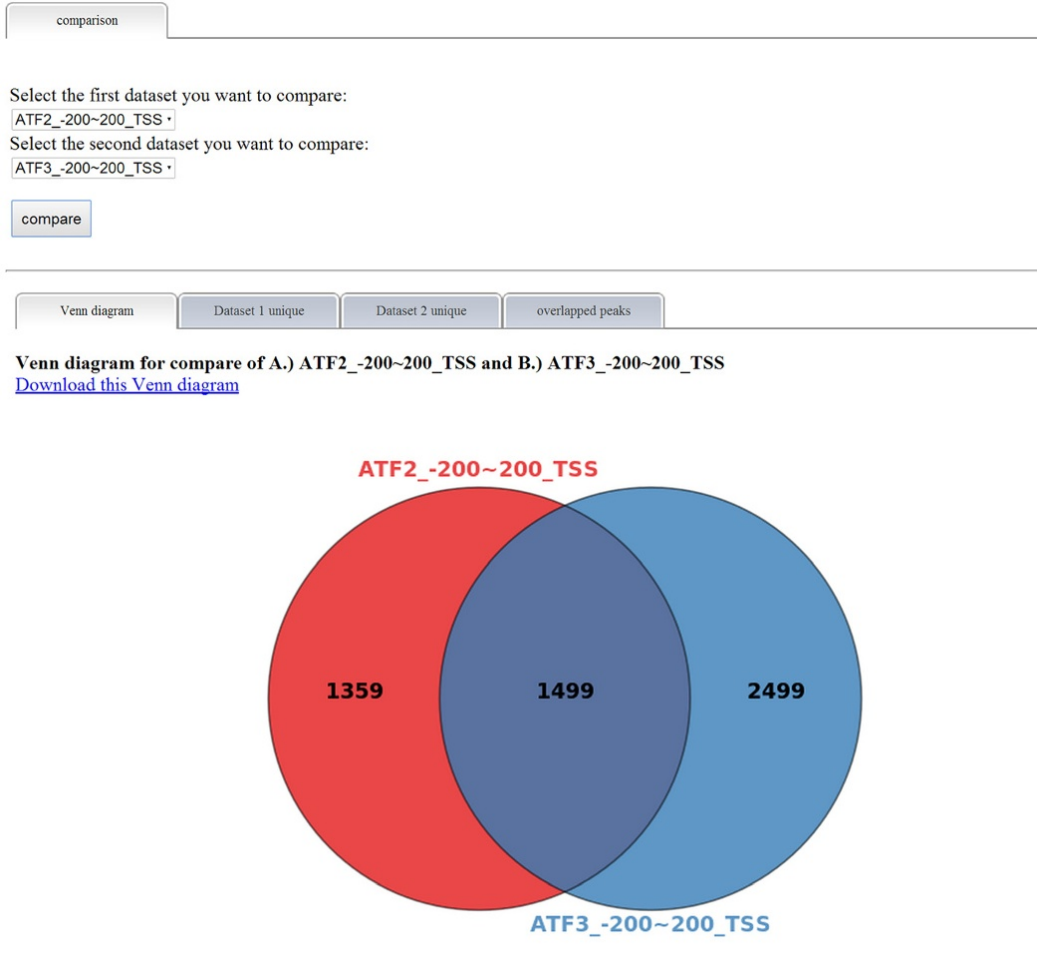
**Comparison between binding sites of ATF2 and ATF3 and Venn diagram.** After selecting ATF2_-200-200_TSS and ATF3_-200-200_TSS for comparison, ChIPseek compares peaks from these two datasets. The overall comparison result is shown as a Venn diagram in the first tab. As shown here, a total of 1,359 peaks are ATF2 unique, 2,499 peaks are ATF3 unique and 1,499 peak pairs are overlapped between the biding sites of ATF2 and ATF3. The detailed peak information for unique peaks and overlapped peak pairs can be found in the following three tabs.

**Figure 5:**
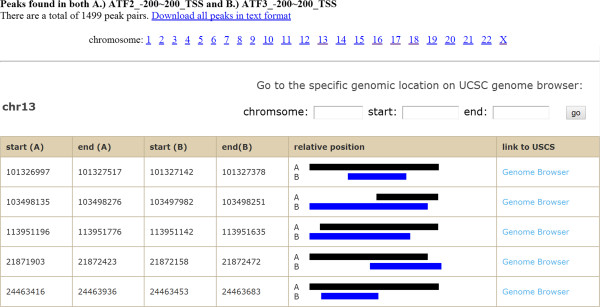
**Overlapped peak pairs.** At the top of this page is shown how many peak pairs are found, and a link is provided to download all peak pairs with their annotation. All overlapped peak pairs are separated into different tables according to their location. As shown here, the table lists peak pairs located on chromosome X. The first four columns list the start and end positions of peaks. The fifth column shows the relative positions of the peak pairs. The last column provides links to the UCSC genome browser for that region of interest. Clicking on the title of each column can sort that column.

ChIPseek further allows peak comparison between two peak files. As a demonstration, we performed a comparison using the two saved subsets of peaks, ATF2_-200-200_TSS and ATF3_-200-200_TSS. In Figure 4, the results of the comparison are displayed in four separate tabs. The first tab is a Venn diagram that shows the number of unique peaks for each dataset and the number of overlapped peaks between the two datasets. Users should note that the overlapped peak section might have redundant peaks. For example, if one peak from ATF3_-200-200_TSS is overlapped with two peaks in ATF2_-200-200_TSS, then they will be counted twice. Therefore, the total number of unique peaks plus overlapped peaks may not necessarily be equal to the total number of peaks in the original datasets. The second and third tabs show tables of unique peaks for each dataset. As shown in Figure 5, the final tab contains tables of various chromosomes with the listed overlapped peaks. In addition to directly comparing the users’ datasets, ChIPseek also includes a database of binding sites for 161 transcription factors generated by ChIP-seq from the ENCODE project [26]. Users may compare their datasets with the experimentally based transcription factor binding sites. As the ENCODE project is focused on human genome, the comparison of transcription factor binding sites is only available for human datasets.

### Demo dataset 2: transcriptional repressor CTCF binding sites

CTCF is known as a DNA methylation-dependent chromatin insulator. Aberrant methylation pattern of the CTCF binding sites of an imprint gene *Igf2/H19* causes the development of Beckwith-Wiedemann Syndrome (BWS) [46–48]. Children with BWS have an over-growth syndrome and predispositions to develop pediatric tumors including Wilms tumor [49]. We downloaded the binding sites of CTCF as our demo dataset. The CTCF dataset is uploaded as a BED file. To demonstrate how to filter the dataset by the length of peaks, we used “Peak length” filter criteria to obtain a subset of peaks that have length range from 100 bp to 150 bp. After that, a subset of 67 peaks out of the original 162,209 peaks is selected and saved. ChIPseek automatically named this subset “CTCF_100-150_len”. The suffix “len” indicates that the peaks in this subset have been filtered according to the peak length.

**Figure 6:**
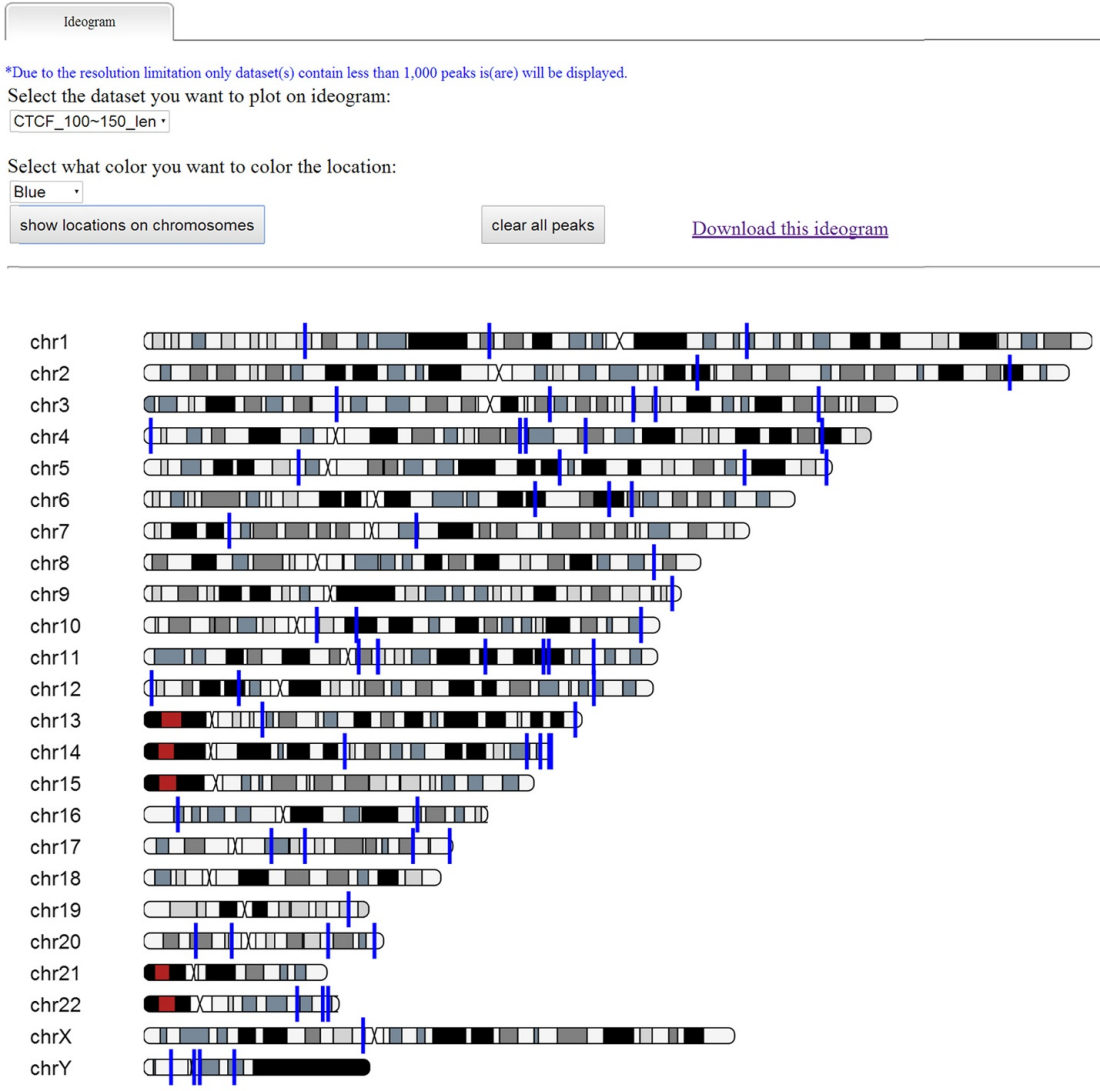
**Chromosome ideograms of CTCF.** Peaks are plotted on the chromosome ideograms at their positions. The information of exact position and nearest gene will appear if the mouse pointer hovers over the peak. Clicking on the peak will link to the UCSC genome browser. There are 10 different colors available for selection: blue, pink, green, yellow, gold, purple, aqua, fuchsia, silver, and red. The user may plot different datasets on the same ideogram with different colors. To plot additional peaks, the user can clear all marks with the “clear all peaks” button.

**Figure 7:**
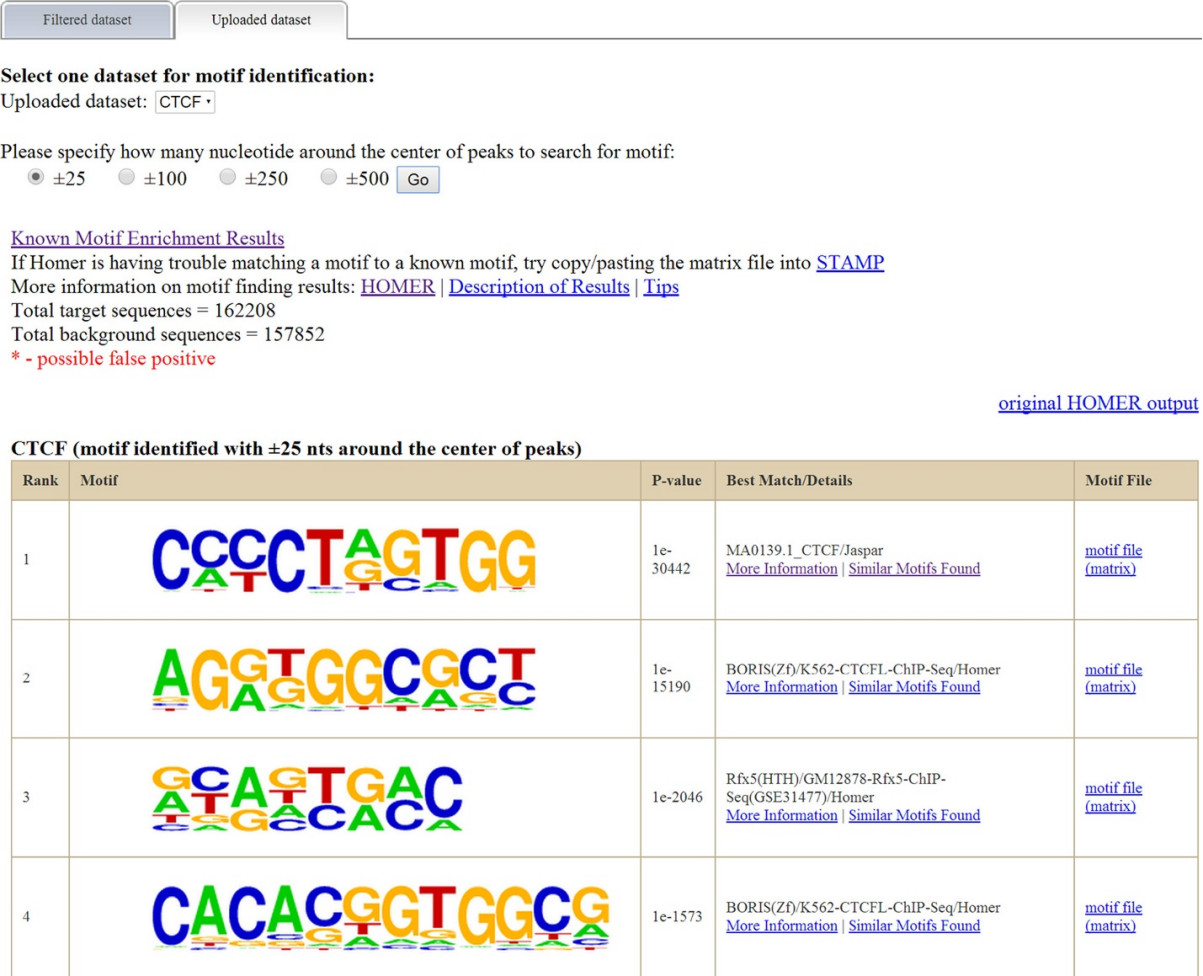
**Motif identification result for binding site of CTCF.** Here is the result of motif enrichment analysis for ±25 bp around the center of CTCF binding sites. The identified motifs are sorted according to their p-values. Clicking on “more information” will display the details for that enriched motif. The original HOMER prediction result is available from the hyperlink provided above the table.

As mentioned in the previous section, users can use multiple filter criteria to narrow down the number of regions that they are interested in. For example if the user want to narrow down the peaks located at promoter regions due to the fact that the transcription factor binding site near the “promoter-TSS” is consider to have a greater biological impact on the activity of the promoter. By selecting peaks located within “promoter-TSS” under the “Peak location” in the filter criteria, the user can filter a total of 11,328 out of 162,209 peaks. On the other hand, if users already have lists of genes which they are interested in, they could also upload lists of genes and filter peaks based on these genes. In addition to use a list of genes for filtering, ChIPseek also offers built-in functional annotations downloaded from GO and KEGG [28, 29] which allow users to generate gene lists based on their interested biological function or pathways. As the annotation functions from GO and KEGG are both constructed in tree structures, users may select interested functions one by one or directly select a whole branch from the annotation tree. It should be noted that, nodes that are belonged to more than one parent, will be listed multiple times under different parents separately in the annotation tree. By allowing this kind of redundancy, ChIPseek can guarantee that the created gene list includes all possible genes related to these selected function groups. Subsets of filtered peaks can be used in further analysis or filtered again by other filter criteria. Users can also remove selected files too if they found some results are not necessary for further analysis.To illustrate the distribution of peaks on chromosomes, users may click “Plot peaks on ideogram” on the menu. After that, users may select the dataset and the color bar (with 10 different colors), and click “show locations on chromosomes”. Due to the resolution limitation, only datasets having less than 1000 peaks can be displayed in this way. Users may display up to 10 different datasets in different colors on the ideograms. In our demo data, dataset “CTCF_100-150_len” is selected, and the locations of 67 peaks are indicated on the chromosomes by blue bars as shown in Figure 6. The chromosome ideogram provides an intuitive way to visualize the overall genomic distribution of all peaks. Currently, the chromosome ideogram is only available for human assemblies (hg17, hg18 and hg19).To identify the enriched motif of CTCF biding sites, users may click on “find motif” in the menu. The users need to specify the range of the enriched motifs. ChIPseek utilizes HOMER for motif identification. According to the user manual of HOMER, 50 bp (±25 bp around the centers of peaks) should be enough to identify the motif for a transcription factor. Alternatively, users may choose 200 bp (±100 bp around the center), which should be enough to find both the primary and “co-enriched” motifs for a transcription factor. As for histone marked regions, ranges from 500–1000 bp (±250- ± 500 bp around the center) may be suitable regions to search. To satisfy all ChIP studies focused on transcription factors, histone modifications and CpG islands, ChIPseek provides four different options: ±25 bp, ±100 bp, ±250 bp and ±500 bp. Users can specify how many nucleotides around the centers of peaks to use as target regions for domain identification according to the users’ experimental purpose. It is worth noting that motif identification is time-consuming and the larger the selected regions the longer it will take to identify the consensus sequences. In our case, because CTCF is a DNA binding protein with known consensus sequence CTCCC, we selected ±25 bp around the center of peaks to search for motifs and the results are shown in Figure 7.

Users should notice that ChIPseek can identify enriched motifs even if they input random data due to the large scale of input data. A significant p-value may be achieved just by random chance. Because ChIPseek uses HOMER to identify potential motifs, ChIPseek only reports motifs that have p-values smaller than 1e-10, which is the cutoff suggested by the HOMER website. However, there may still be some false positives after this filter. Therefore, it is still very important for the users to determine which patterns may truly be recognized by the binding protein. Users may identify most promising candidates by checking the p-value distribution and setting a customized p-value cutoff. As ChIPseek shows p-values in ascending order, the p-value followed by another dramatically increased p-value may be the proper cutoff. Another clue for candidate motif selection is that different offsets of a true motif may appear in the report several times. For example, in Figure 7, the pattern “GGTGG” shows up several times at the top four sequence logos. Therefore, this GGTGG is likely to be part of a real motif. As the binding consensus sequence of CTCF in the human genome has already been investigated in a previous study [50], we found this pattern to indeed be present in the consensus binding sequences of CTCF. Meanwhile, many motifs have been identified, and their consensus sequences are well known. ChIPseek also uses HOMER to perform motif enrichment analysis for those known motifs. Users may also explore known motifs by following the hyperlink “Known Motif Enrichment Results” above the table.

## Conclusions

Here we present ChIPseek as a web tool for analyzing ChIP-seq and ChIP-chip data. By integrating HOMER and BEDTools, ChIPseek is an easy-to-use software package for the first-time user with only basic computer skills who wants to analyze ChIP data. ChIPseek guides the users step-by-step to obtain the peak annotation, locations, sequences, and useful statistics as charts and histograms for visualizing the properties of the peaks. ChIPseek also provides UCSC genome browser links so that the users can investigate peaks further. A unique feature of our tool is that it includes filter tools which allow users to select interested peak subsets based on peak lengths, distance to the nearest TSS, peak location, user uploaded gene lists or genes belong to user selected functions. For human datasets, users may also use ChIPseek to plot peaks on an ideogram, which offers an overview of genomic distribution across different chromosomes. When comparing two datasets, ChIPseek generates a Venn diagram, lists of unique peaks for each dataset and overlapped peak pairs with their relative positions. We also showed that ChIPseek can help users identify enriched binding motifs for transcription factors and DNA binding protein factors. In addition to these convenient analysis tools, ChIPseek also has a built-in database of human transcription factor binding sites available for comparison. In summary, ChIPseek includes many desired functions that are available through an intuitive user interface. ChIPseek is a free, web-based service. The use of ChIPseek requires no knowledge of Linux, no programming, no installation and no login. The ChIP data analysis tools and databases provided by ChIPseek will offer invaluable assistance for biologists whose studies focus on protein-DNA interaction, gene transcription regulation, epigenetics, chromatin organization and cancer biology.

### Availability and requirements

ChIPseek is freely available at http://chipseek.cgu.edu.tw and all the source codes of pipelines and parsers are also available upon request. Currently, ChIPseek can support most commonly used browsers such as Chrome, Firefox, Internet Explorer and Safari.

## Abbreviations

ChIP: Chromatin immunoprecipitation
TSSs: Transcription start sites
bp: Base pair
UTR: Untranslated region
TTS: Transcription termination site
BWS: Bechwith-Wiedemann Syndrome.
